# Influence of plasmon excitations on atomic-resolution quantitative 4D scanning transmission electron microscopy

**DOI:** 10.1038/s41598-020-74434-w

**Published:** 2020-10-21

**Authors:** Andreas Beyer, Florian F. Krause, Hoel L. Robert, Saleh Firoozabadi, Tim Grieb, Pirmin Kükelhan, Damien Heimes, Marco Schowalter, Knut Müller-Caspary, Andreas Rosenauer, Kerstin Volz

**Affiliations:** 1 Materials Science Centre and Department of Physics, Philipps University Marburg, Hans-Meerwein-Straße 6, 35032 Marburg, Germany; 2 Institut für Festkörperphysik, Universität Bremen, Otto-Hahn-Allee 1, 28359 Bremen, Germany; 3 Ernst-Ruska-Centre for Microscopy and Spectroscopy with Electrons, Forschungszentrum Jülich, Wilhelm-Johnen-Straße, 52425 Jülich, Germany; 4 2nd Institute of Physics, RWTH Aachen University, Templergraben 55, 52062 Aachen, Germany

**Keywords:** Materials science, Nanoscience and technology, Physics

## Abstract

Scanning transmission electron microscopy (STEM) allows to gain quantitative information on the atomic-scale structure and composition of materials, satisfying one of todays major needs in the development of novel nanoscale devices. The aim of this study is to quantify the impact of inelastic, i.e. plasmon excitations (PE), on the angular dependence of STEM intensities and answer the question whether these excitations are responsible for a drastic mismatch between experiments and contemporary image simulations observed at scattering angles below $\sim$ 40 mrad. For the two materials silicon and platinum, the angular dependencies of elastic and inelastic scattering are investigated. We utilize energy filtering in two complementary microscopes, which are representative for the systems used for quantitative STEM, to form position-averaged diffraction patterns as well as atomically resolved 4D STEM data sets for different energy ranges. The resulting five-dimensional data are used to elucidate the distinct features in real and momentum space for different energy losses. We find different angular distributions for the elastic and inelastic scattering, resulting in an increased low-angle intensity ($\sim$ 10–40 mrad). The ratio of inelastic/elastic scattering increases with rising sample thickness, while the general shape of the angular dependency is maintained. Moreover, the ratio increases with the distance to an atomic column in the low-angle regime. Since PE are usually neglected in image simulations, consequently the experimental intensity is underestimated at these angles, which especially affects bright field or low-angle annular dark field imaging. The high-angle regime, however, is unaffected. In addition, we find negligible impact of inelastic scattering on first-moment imaging in momentum-resolved STEM, which is important for STEM techniques to measure internal electric fields in functional nanostructures. To resolve the discrepancies between experiment and simulation, we present an adopted simulation scheme including PE. This study highlights the necessity to take into account PE to achieve quantitative agreement between simulation and experiment. Besides solving the fundamental question of missing physics in established simulations, this finally allows for the quantitative evaluation of low-angle scattering, which contains valuable information about the material investigated.

## Introduction

Progress in both the fundamental understanding of solid state physics and the characterisation of materials down to the atomic scale is currently stimulated drastically by increasing the dimensionality of experimental data. A decade ago, Z-contrast scanning transmission electron microscopy (STEM) imaging has been lifted to a quantitative level which allows to measure the chemical composition at atomic resolution^1–6^ by utilizing the high-angle scattering of electrons captured with an annular dark field (ADF) detector, and the comparison with extensive simulations. Even more recently, the availability of ultra-fast cameras^7–11^ operating at many thousands of frames per second and other dedicated setups^12^ allow the recording of large up to four-dimensional data sets providing high sampling of both real and reciprocal space simultaneously, referred to as “four-dimensional STEM” (4D STEM). Besides enabling efficient STEM phase contrast imaging^13–15^, employing momentum-resolved STEM for an angular multi-range analysis to simultaneously measure, e.g., the local content of multiple chemical elements, lattice strain and the specimen thickness was envisaged^12^. In particular, this relies on the assumptions that a certain set of specimen parameters yields a unique diffraction pattern within the boundary of available case-specific prior knowledge, and that simulation methods exist which resemble the experimental conditions and scattering physics accurately at all scattering angles. As a matter of fact, such a comprehensive characterisation of functional devices in the fields of optoelectronics, energy and information technology would not only give insight into structure-property relationships and drastically enhance the visibility of light elements by electron microscopy, but also provide an important feedback loop for, e.g., the epitaxy of functional nanostructures.

Interestingly, the latest access to the full distribution of electron scattering in experiments has rather caused the stagnation of quantitative STEM than its extension. In particular, dramatic discrepancies of up to a factor of two between experimental low-angle intensities and thorough simulations employing the most recent methods have been observed^12^. Since relativistic, dynamical scattering of electrons including quasi-elastic scattering at phonons is incorporated accurately within contemporary quasi-elastic multislice simulations in frozen phonon approximation^16–22^, the dominant hypothesis ascribes the mismatch to further inelastic scattering caused by plasmon or core excitations. Nevertheless, only little experimental evidence and theory have supported this interpretation so far^23,24^, despite its key role concerning the fundamental physics of relativistic low-angle scattering, and its untapped potential for fully quantitative STEM employing the whole angular range of electron scattering. However, potential factors other than inelastic scattering, which may contribute to the low-angle intensities, should be mentioned here as well, e.g. if phonons in the simulations are described by a correlated or uncorrelated movement^18,20,25^, the correct choice of atomic scattering potentials^13,26–28^ or the presence of amorphous layers from sample preparation^29–31^.

Following the approach of multidimensionality, this study introduces the dependence on energy loss suffered by the electrons passing the specimen in addition to momentum and spatial resolution to obtain a five-dimensional data set. This is achieved by using a dedicated experimental setup where an ultra-fast pixelated camera is mounted behind an energy filter in an aberration-corrected STEM in addition to another setup with a conventional camera behind the energy filter. These two setups are representative for systems which are used for quantitative STEM^1–6^. For two well-defined material systems, we demonstrate that inelastic scattering, predominantly the excitation of plasmons, is responsible for a redistribution of low-angle scattering in diffraction space and hence leads to the mismatch between contemporary theories for quasi-elastic scattering and non-energy-filtered STEM. To this end, we use the technologically relevant semiconductor silicon (Si) in [010] projection and metallic platinum (Pt) imaged in [110] direction to study the impact of inelastic scattering in a high-Z metal for which it is expected to be even more important. Further, the capability of approaches to incorporate inelastic processes into frozen-phonon multislice simulations is investigated, balancing computational effort and accuracy. This leads to possible routes forward to enable the quantitative interpretation of low-angle scattering so as to unfold the full potential of momentum-resolved STEM for materials analysis.

## Results

As a starting point, distinct angular features for the different energy-loss ranges will be elaborated. The dependence of the findings on the thickness of the sample will be discussed. In a second step, the experimental findings will be compared to simulations, in order to approach the impact of plasmon excitations from the theoretical point of view. The impact of the plasmon excitations on low scattering angles will be highlighted by employing simulated as well as experimental atomic resolution ADF images generated for different angular regimes. Thirdly, spatial resolution present in the data sets will be exploited further by evaluating the amount of inelastic scattering with respect to the distance of the STEM probe to an atomic column. Finally, the potential impact of inelastic scattering on momentum-resolved measurements will be discussed.

### Angular dependencies of elastic and inelastic scattering

To explore the influence of inelastic scattering on low-angle STEM image intensities, we investigate the angular dependencies of zero-loss scattering, which includes elastic scattering and quasi-elastic phonon scattering, on the one hand and inelastic scattering with significant energy losses, due to plasmon excitations, on the other hand. In this study, the focus is put onto plasmon losses. We want to point out that, if the angular distributions of the elastic and inelastic signals were identical, there would be no impact on the STEM intensity at all, because the total measured intensity in any angular range would be the same, no matter if an impinging electron had transferred any energy to the sample or not.

With the first experimental setup, i.e. a Titan 80/300 TEM operated at 300 kV, energy filtered diffraction patterns are recorded for five different thicknesses of a Si [010] specimen using a probe semi-convergence angle of 9 mrad. Each diffraction pattern is accumulated on-the-fly, while the probe scans over a region of several nm$^2$, accordingly the resulting patterns will be referred to as position-averaged convergent beam electron diffraction patterns (PACBED)^32^.

**Figure 1:**
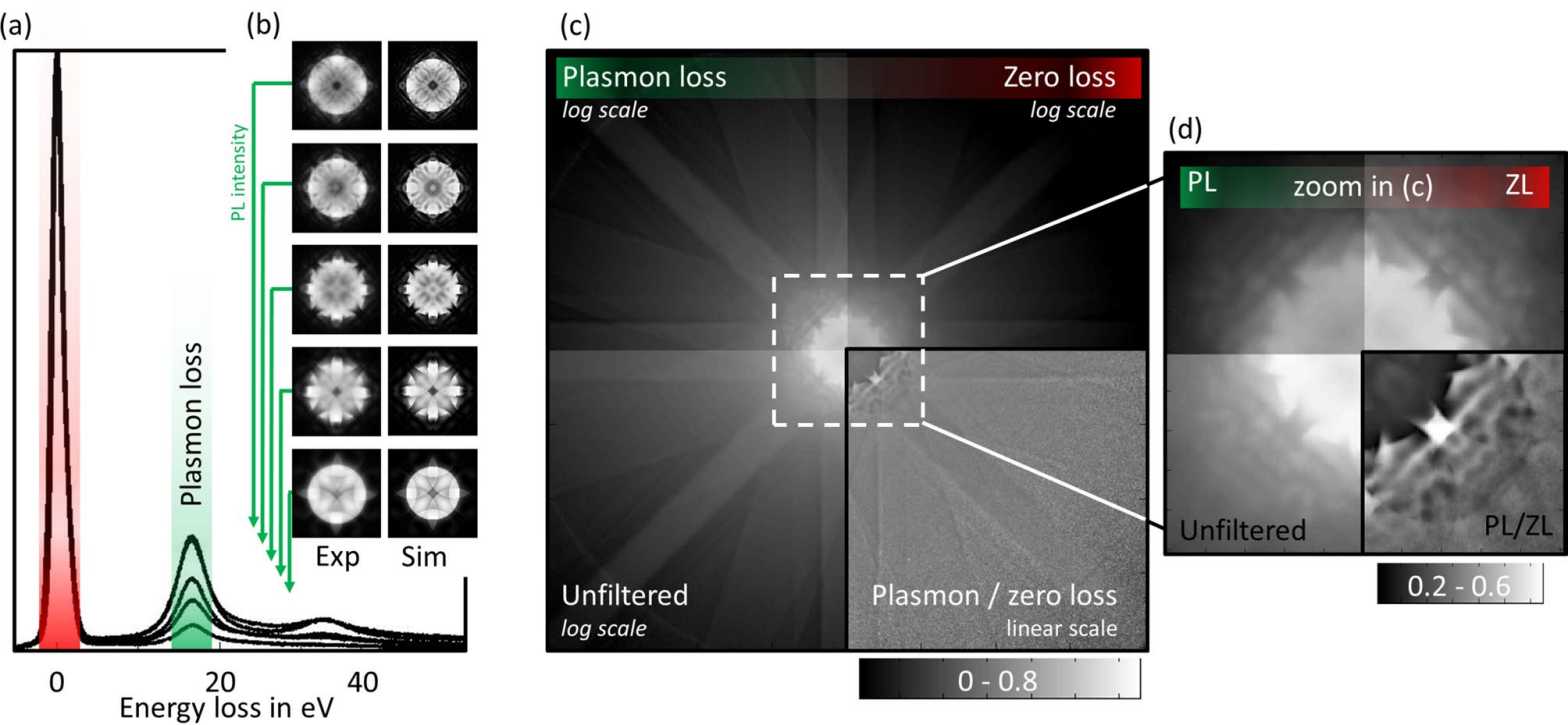
(**a**) EEL spectra and (**b**) experimental and simulated PACBEDs for five different specimen thicknesses. (**c**) Example for a PACBED used to obtain angular scattering intensities. The four quadrants show energy filtered diffraction patterns (plasmon loss, zero loss and unfiltered) using the same logarithmic scale as well as the ratio of plasmon-loss pattern and zero-loss pattern with linear scale. (**d**) Magnification of the inner part with adjusted scaling to improve the visibility of the details in the center of the pattern.

Figure 1a shows experimental electron energy-loss (EEL) spectra for all thickness steps. The intensity of each spectrum is normalized to the maximum intensity of the zero-loss peak. Besides the zero-loss peak, the spectra are dominated by the plasmon-loss peak at $16.7\,\mathrm {eV}$, which increases with thickness. The thicknesses are determined using a comparison of experimental PACBED patterns with simulations^31,32^, both of which are shown in Fig. 1b. The resulting thicknesses are 30, 55, 85, 115 and $140\,\mathrm {nm}$ ($\pm \,5\,\mathrm {nm}$). Although this study is motivated by the fact that quantitative interpretation of low-angle scattering is still not possible, it should be noted that high-angle ADF intensities (HAADF) can be used for quantitative thickness determination of Si [010] as shown in Refs. ^4,31^. Indeed, quantitative HAADF^2,4,6,31,33,34^ gives thicknesses of 30, 60, 85, 110 and $130\,\mathrm {nm}$ ($\pm \, 5 \,\mathrm {nm}$) that are in excellent agreement with the thicknesses obtained from PACBED within the uncertainty of the methods.

For each thickness, diffraction patterns are recorded without energy filter (unfiltered) and with a 10 eV energy slit centered around $0\,\mathrm {eV}$ (zero loss) as well as around 16 eV (plasmon loss). In Fig. 1c these three diffraction patterns are shown in three of the quadrants for the sample area with $85\,\mathrm {nm}$ thickness.

It becomes obvious from the inner parts of the diffraction patterns shown magnified in Fig. 1d that the plasmon-loss pattern resembles a blurred version of the zero-loss pattern. The ratio of the plasmon-loss pattern and the zero-loss pattern is shown in the fourth quadrant (bottom right) of Fig. 1c,d. It indicates that the excitation of plasmons leads to a redistribution of intensity from the central beam (lower ratio) towards the outer region (increased ratio).

**Figure 2:**
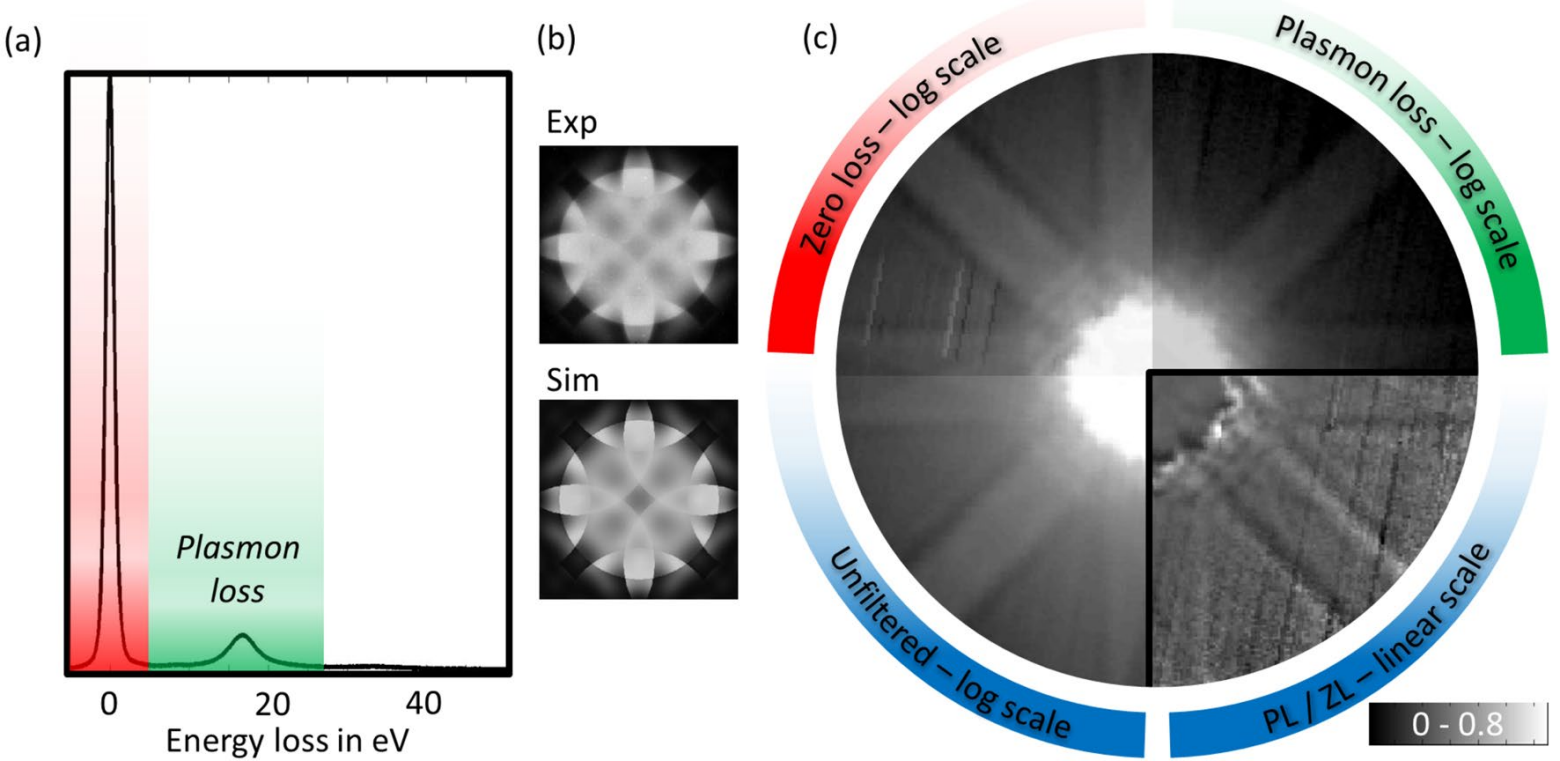
(**a**) EEL spectrum and (**b**) experimental and simulated PACBEDs for a specimen thicknesses of 42 nm. (**c**) Synthetic PACBEDs generated from a 4D data-set. The four quadrants show energy filtered synthetic PACBEDs (plasmon loss, zero loss and unfiltered) on a common logarithmic intensity scale as well as the ratio of plasmon-loss and zero-loss pattern on a linear scale.

The measurements presented demonstrate the drastic impact of energy loss on the angular distribution of scattering in reciprocal space, but do not provide spatial information in real space yet, since each PACBED is a positional average of the field of view of several nm$^2$. In order to investigate the angular dependencies at atomic spatial resolution and provide more versatile analysis schemes, additional 4D data-sets are acquired selecting the distinct regions of the energy spectrum with the slit of the energy filter in a second experimental setup. To this end, a $C_S$-corrected JEOL JEM 2200 FS operating at 200 kV with a probe semi-convergence angle of 15.1 mrad is used. The fast readout of 1000 fps of the attached pnCCD allows for the acquisition of a full diffraction pattern at each scan point. To show the influence of this quite different experimental conditions on the angular distributions, in a first step, synthetic PACBED patterns are generated from these 4D data-sets by averaging over all 256 $\times$ 256 individual diffraction patterns acquired while scanning over the field of view of 4 $\times$ 4 nm. Here, electrons in the energy interval between − 5 and 5 eV are used to generate the zero-loss signal, whereas energies from 5–27 eV are used to generate the plasmon-loss signal. This comparably wider energy window ensures that enough electrons which experienced plasmon loss are detected within the 1 ms exposure of a single frame. In analogy to Figs. 1a, 2a shows the experimental EEL spectrum of a 42 nm thick Si sample, the sample thickness is again determined by PACBED and quantitative STEM carried out separately under the same conditions as used for the PACBEDs in Fig. 1b. Figure 2b depicts a complementary PACBED pattern of the same sample region alongside the corresponding simulation for a thickness of 42 nm.

Figure 2c displays the synthetic PACBED patterns generated from the unfiltered data, the zero-loss data and the plasmon-loss data on a common logarithmic intensity scale in analogy to Fig. 1c,d. The experimental camera length is chosen to record a maximum scattering angle of $\sim$ 60 mrad, to adequately sample the region of interest with the limited amount of pixels of the pnCCD in comparison to the conventional CCD used for acquisition of the PACBED. To a good approximation, the total intensities of the zero-loss set (64% of impinging beam) and the plasmon-loss set (19%) add up to the one of the unfiltered set (85%). This suggests that, at least for this sample thickness and angular range, higher energetic features, i.e. a second plasmon or core loss, can safely be neglected.

Comparing the individual patterns, it becomes obvious that the plasmon-loss pattern appears blurred in comparison to the zero-loss pattern, in agreement with the PACBED acquired at way different experimental conditions, e.g. 300 kV instead of 200 kV. For example, the interference fringes in the 202 discs are more prominent in the zero-loss data set compared to the plasmon-loss pattern. The ratio of the plasmon-loss pattern and the zero-loss pattern is shown in the fourth quadrant (bottom right) of Fig. 2c. It once more indicates that the excitation of plasmons leads to a redistribution of intensity from the central beam towards the outer region. In total, there is very good qualitative agreement between the synthetic PACBEDs generated from the 4D data-sets and the PACBEDs presented in Fig. 1. This highlights the generality of the features found, since they can be observed under very different experimental conditions, e. g. incident beam energies and semi-convergence angles.

To investigate this angular distribution in a quantitative manner, the intensities of both, the PACBEDs recorded at 300 kV and the synthetic PACBEDs recorded at 200 kV, are azimuthally averaged. Such angular intensity profiles allow for the comparison to established ADF techniques using integrating ring-shaped detectors. These profiles can contain valuable information on thickness, composition, disorder or strain within a sample^12,35–37^.

**Figure 3:**
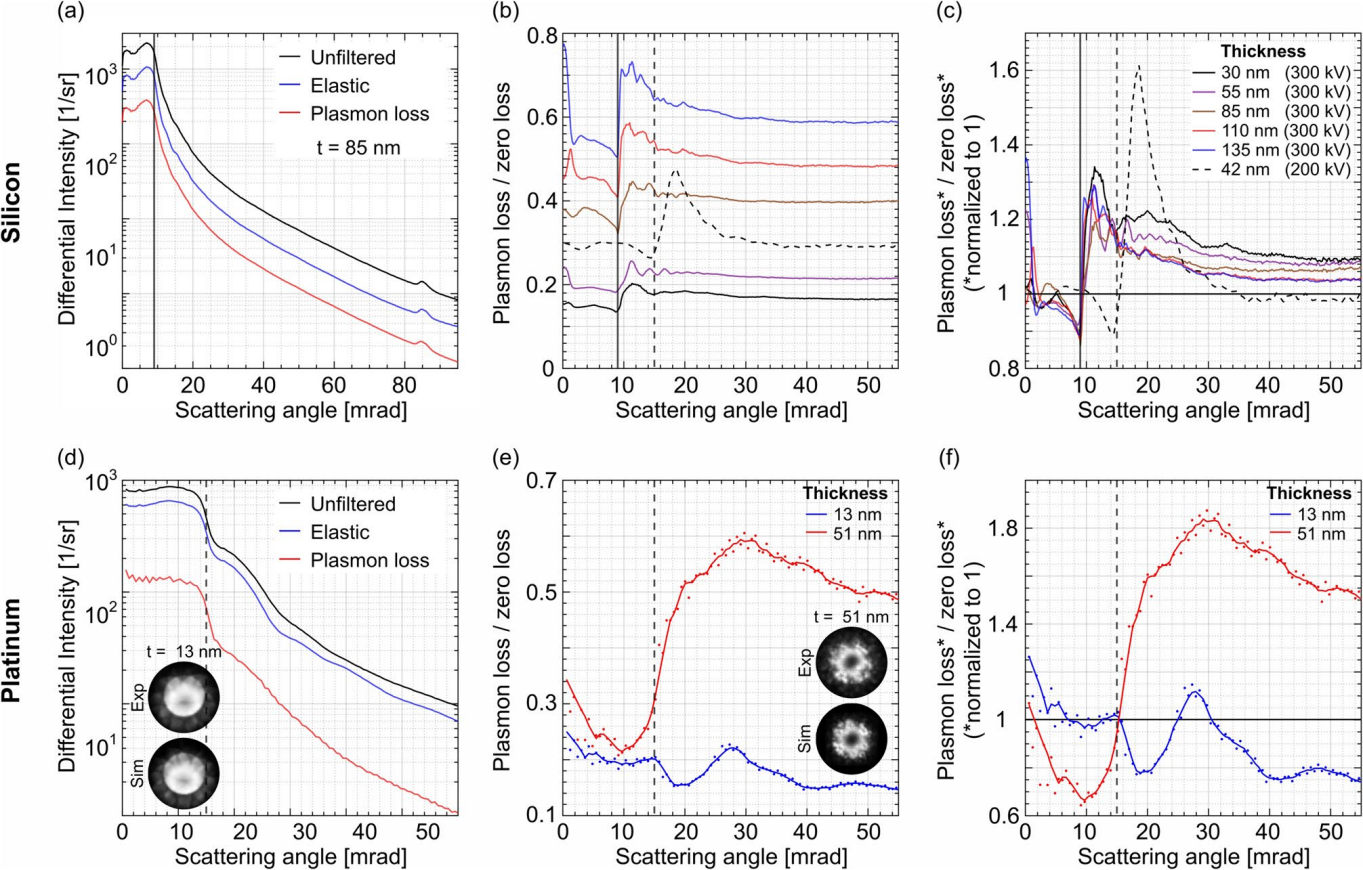
Angular distribution of the scattering for different thicknesses: (**a**) shows differential intensity per solid angle plotted over scattering angle obtained by azimuthal averaging of energy-filtered diffraction patterns (example shown for Si with a thickness of $85\,\mathrm {nm}$) Black: unfiltered; blue: zero loss; red: plasmon loss. (**b**) Displays the ratios of plasmon-loss and zero-loss intensities for all thickness steps in the Si, while (**c**) shows these ratios after a normalisation of both signals as described in the text. (**d**) Shows the differential intensity plotted over scattering angle for Pt with a thickness of $13\,\mathrm {nm}$. Black: unfiltered; blue: zero loss; red: plasmon loss. In (**e**) the ratio of plasmon-loss and zero-loss intensities for two thicknesses are displayed, while (**f**) shows the normalized ratios. The vertical lines in each plot represent the semi-convergence angles used at 300 kV (solid line) and 200 kV (dashed line), respectively.

The angular intensities derived from the different Si data sets are presented in Fig. 3a–c. Solid lines belong to the measurements at 300 kV and dashed lines to the one at 200 kV. Vertical lines of the same style mark the corresponding semi-convergence angle used Fig. 3a depicts the differential intensities per solid angle derived from the unfiltered (black line), the zero-loss (blue line) and the plasmon-loss PACBED (red line), respectively, for 85 nm specimen thickness (300 kV). These are the very patterns which are shown as an example in Fig. 1c,d. Since the differences of the angular distributions of the elastic and inelastic intensity are of special interest, the ratio of the angular intensities of plasmon loss and zero loss is calculated, which is shown in Fig. 3b.

The ratio curves derived from the thickness series acquired at 300 kV shift to higher values with increasing specimen thickness as the inelastic contribution increases, whereas the elastic intensity decreases. It should be noted that the energy slit of $10\,\mathrm {eV}$ width does not collect all intensity within the broad plasmon peak resulting in a larger apparent mean free path (MFP) of approximately $300\,\mathrm {nm}$ for the plasmon-loss signal. In contrast, the reduction of the zero-loss intensity corresponds to a MFP close to the expected value of $~180\,\mathrm {nm}$^38^, since the whole intensity is collected by the energy window used. The analogue graph derived from the 4D data-set acquired at 200 kV from a region with a thickness of 42 nm is shown as dashed line. Since the MFP differs from the one of the other measurements, due to the different primary energy and the wider energy window used, the graph does not align into the observed order of thicknesses, instead it is situated between the graphs for 55 and 85 nm thickness.

In conclusion, all experimental results in Fig. 3b allow the following qualitative statements: For all thicknesses, the curves have in common that the ratios are comparably low below the semi-convergence angle of the probe, which is $9\,\mathrm {mrad}$ and $15.1\,\mathrm {mrad}$, respectively. For scattering angles slightly larger than the semi-convergence angle, the ratios increase to a maximum and show a smooth decay in the further course. Above $\sim \,40\,\mathrm {mrad}$, i.e. $25{-}30\,\mathrm {mrad}$ above the semi-convergence angles, the ratios adopt a constant level. This means for the two semi-convergence angles investigated here, the angular dependencies of elastic and inelastic electrons are identical for angles above $\sim \,40\,\mathrm {mrad}$, which is the reason why experimental and simulated intensities in the HAADF regime agree perfectly, even if plasmon excitation is neglected. But for higher semi-convergence angles the effects of inelastic scattering may extend to even higher angles.

To be able to directly compare this behaviour for different thicknesses, Fig. 3c shows the ratios of the plasmon-loss and zero-loss intensities, but here each individual signal has been normalised to unity beforehand. This representation is almost independent of the MFPs as the thickness dependence of the total scattered intensity is completely eliminated from both signals. The graphs reveal that these ratios indeed show a similar behaviour for all thicknesses: The inelastic intensity is reduced with respect to the zero-loss intensity below the semi-convergence angle of the probe and it is increased for angles higher than that. This has to be interpreted as the result of a redistribution of scattered electrons towards higher scattering angles. Even in the range between 30 mrad and 40 mrad, the curves are not constant. Therefore, plasmon excitation transfers intensity into angles that are much higher than one might expect, considering that the characteristic scattering angles for this plasmon excitation are $23\,\upmu {\hbox {rad}}$ and $34\,\upmu {\hbox {rad}}$ for 200 and 300 kV, respectively^39^.

This highlights that elastic and inelastic scattering have significantly different angular dependencies. Consequently, neglecting the plasmon excitations in the accompanying simulations can indeed lead to the observed underestimation of the experimental intensity in the angular range below $\sim$ 40 mrad. If this discrepancy could be overcome by considering plasmon excitations, it would finally allow for the quantitative evaluation of the whole angular range of electron scattering in STEM.

So far, the influence of plasmon excitations was investigated for one material only. However, the impact of plasmon excitations may be material dependent, since important parameters such as e.g. the MFP, Bragg angles and structure factors differ. Therefore, the angular ranges in which a contribution of plasmon excitations is expected may vary. To elucidate this, we choose Pt ($Z=78$) as a complementary example since it is a rather heavy element compared to the comparably light Si ($Z=14$). Moreover, Pt is a metal, whereas Si is a semiconductor.

Two regions of interest with thicknesses of 13$\,\pm \,5$ and 51$\,\pm \,5$ nm are analysed in [110] direction. 4D STEM data are recorded at atomic resolution without energy filtering, in addition with a 10 eV wide energy window centered around zero loss, and a 30 eV wide energy window centered around the first plasmon peak at $\sim$ 22.6 eV. The thickness is again evaluated using the comparison between the zero-loss filtered diffraction patterns and an elastic PACBED simulation^40^.

Figure 3d depicts the differential intensities derived at the thinner, i.e. 13 nm thick, region. The color coding is the same as for the Si case, i.e. unfiltered, zero-loss and the plasmon-loss signal are shown as black, blue and red line, respectively. The corresponding experimental and simulated PACBED patterns are shown as insets in Fig. 3d,e, respectively. The PACBED pattern derived from the thinner region reveals a slight mistilt of $\sim$ 5.2 mrad with respect to the [110] zone-axis.

In analogy to the Si data, the plasmon signal (red line) appears blurred in comparison to the zero-loss signal (blue line). This can be seen, for instance, from the absence of intensity undulations caused by averaging over diffraction discs in the plasmon signal, which in contrast are visible in the zero-loss signal. The plasmon-loss/zero-loss ratio is plotted in Fig. 3e for both sample thicknesses. Due to the rather small energy window used for the plasmon-loss signal, the ratios for the Pt material appear slightly noisier than the ones for the Si. To accommodate for this, the ratios are smoothed via a gliding average. From the observation of the scattering characteristics, a few remarks can be made, echoing the findings from the Si case study. As expected, the overall proportion of inelastically scattered electrons increases with specimen thickness, i.e. in the thin region, 73% of all electrons are in the zero-loss window, while this number decreases to 39% in the thicker region. The course of both graphs is very similar to the ones of Si, i.e. lower ratios at angles below the semi-convergence angle followed by a region of elevated ratio followed by a smooth decay and finally adopting a constant value. These constant values are higher for Pt than for Si with a comparable thickness, reflecting the higher Z of the Pt and the resulting higher contribution of inelastic scattering. The additional drop of the ratio at around 20 mrad observable for the thinner region is most likely caused by the mistilt mentioned above, leading to artefacts during azimuthal averaging.

Figure 3f shows the corresponding normalized ratios. As in the Si case, the graphs reveal the same qualitative behaviour for both thicknesses, i.e. the inelastic intensity is reduced with respect to the zero-loss intensity below the semi-convergence angle and it is increased for angles higher than that.

In total, the observed angular features are comparable for both materials and for two different experimental setups, this highlights the general importance of plasmon excitations in all techniques involving low-angle scattering.

### Simulation of plasmon excitations in electron scattering

With the intention to include the observed plasmon-interaction effects into frozen-phonon multislice simulations, the STEMsim software package^19^ is extended. Plasmon excitations are included by using a transition potential, which is represented by

1 $$\begin{aligned} {\mathscr {F}} \left\{ V_{\text {TP}}({\vec{r}}) \right\} =V_{\mathrm {TP}}({\vec{k}}) \propto \sqrt{\frac{1}{\sqrt{\omega (k)}} \frac{1}{{k_z^2 + k^2}}} \end{aligned}$$

with the bulk plasmon dispersion relation $\omega (k)$ and the characteristic wave vector $k_z$^39,41–43^, which is proportional to the plasmon energy-loss. The implementation details shall be discussed in a later publication, but the basic scheme is the following: The transition potential is applied in real space by multiplication by the wave function. It allows for the transition into an inelastic channel, in which consecutive elastic propagation follows. Additionally, the transition potential is shifted to various positions inside the specimen, the signals of which are then added up incoherently. This ensures the incoherence of plasmon excitations belonging to different wave vectors. The plasmon dispersion relation $\omega (k)$ is derived following the self-consistency model from Ref.^44^, but calculated numerically without an approximating series expansion.

**Figure 4:**
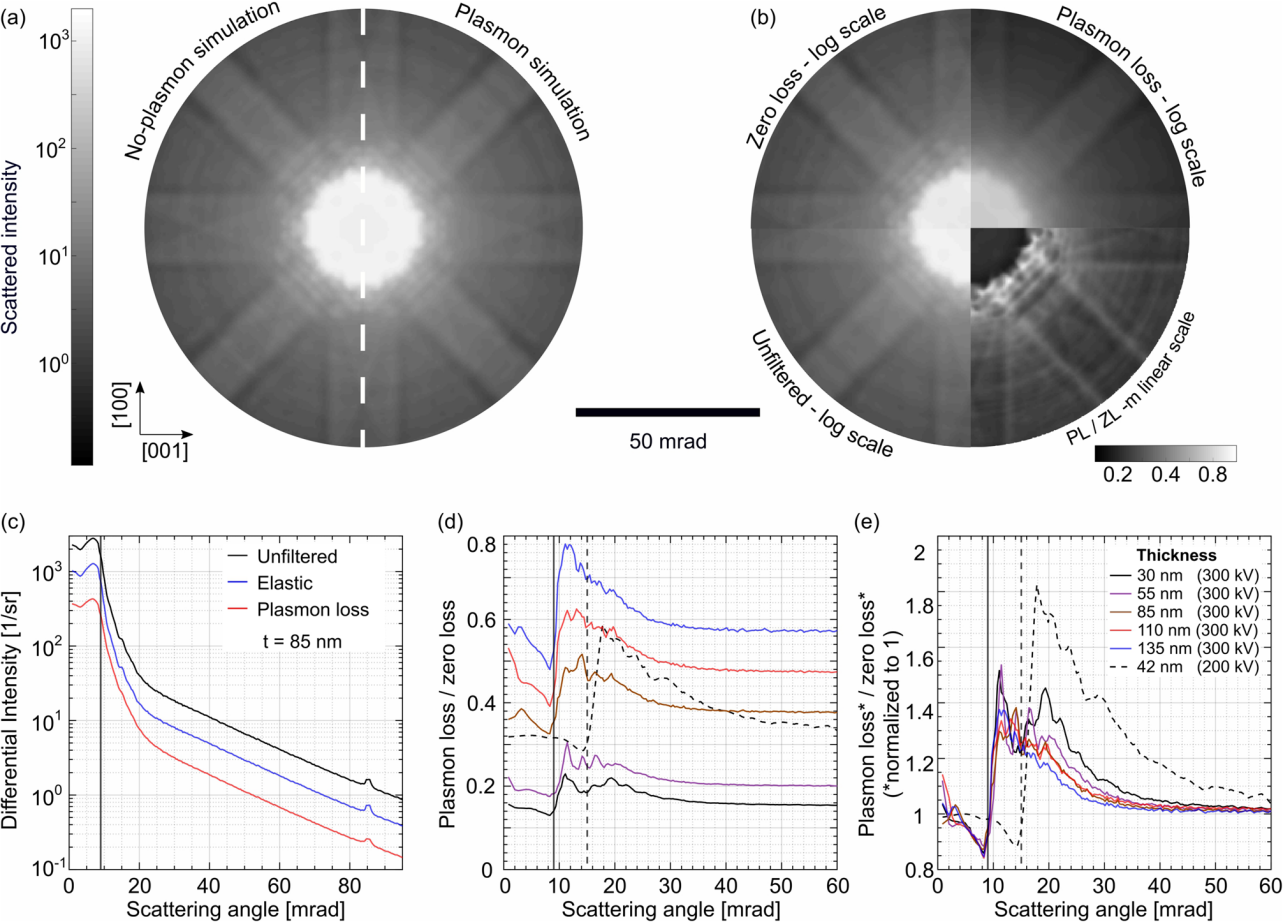
Simulated synthetic PACBED patterns of Si [010] with 42 nm thickness (200 kV) (**a**) neglecting (left) and including plasmon excitations (right). The two components of the latter one, i.e. plasmon-loss part and zero-loss part, are shown as quadrants in (**b**) and their ratio as another one. The simulated differential intensities for the 85 nm thick data set (300 kV) are collected in (**c**) (Black: unfiltered; blue: zero loss; red: plasmon loss). The plasmon-loss/zero-loss ratios and the corresponding normalized ratios for different thicknesses are depicted in (**d**) and (**e**), respectively (Solid lines: 300 kV; dashed lines: 200 kV). The corresponding experimental curves for a thickness of 85 nm are shown again for comparison reasons as dotted lines in (**d**) and (**e**).

This concept is then used for the incorporation of plasmon excitations into the simulations for the Si material system, using the parameters of the experiments conducted. The MFP is chosen according to the width of the energy slit. The characteristics of the cameras are included by means of the modulation transfer functions (MTF), which were determined with a modified knife-edge method^45,46^. Further details are given in the methods section, the results are displayed in Fig. 4.

The simulated synthetic PACBED pattern derived from a Si super cell with a thickness of 42 nm (i.e. 77 unit cells) is shown in Fig. 4a. The left-hand side of the pattern is generated without the consideration of plasmon excitations, whereas in the pattern on the right-hand side, plasmon excitations are taken into account. In analogy to Fig. 2, the individual contributions of the pattern, i.e. the zero-loss part and the plasmon-loss part, are shown as quadrants in Fig. 4b on the same logarithmic intensity scale. The ratio of plasmon-loss/zero-loss is shown on a linear intensity scale as fourth quadrant. The simulation parameters are chosen to resemble the experimental ones used for the acquisition of the 4D data-sets presented in Fig. 2c, e.g. 200 kV and 15.1 mrad semi-convergence angle. The main features observed in the experimental patterns, e.g. the blurred plasmon-loss signal, are well resembled by the simulation. Moreover, the simulated ratio shows the distinct low values in the central disc and increased values outside which are observable in the experiments.

Figure 4c–e display the simulated differential intensities in the same manner as the experimental data in Fig. 3. The dependencies of the plasmon-loss/zero-loss ratios collected for the different thicknesses in Fig. 4d resemble the experimental curves (Fig. 3a–c) very well: Especially the same increase of intensity in the low-angle regime up to 30 mrad at the expense of the central beam as measured is well resembled, which can be seen in more detail in the normalized ratio plots depicted in Fig. 4e, which also reveals the similarity of all thicknesses except for the thinnest area, as it was the case with the experimental data. To allow for easy comparison to the experimental data, the corresponding experimental curves for a thickness of 85 nm are shown once more as dotted lines in Fig. 4d,e.

Even though no perfect agreement on a quantitative level is achieved yet, the simulations are able to reproduce the observed features and qualitatively agree very well with the experiment. Potential factors explaining the remaining discrepancies will be discussed in a later section, where the actual impact of the plasmon excitations on ADF intensity is discussed as well. The intensity redistribution observed is surprising given the small characteristic angle of $34\,\upmu {\hbox {rad}}$, which corresponds roughly to the width of the transition potential. One should realise that the simulated plasmon diffraction pattern as indicated above can be formally expressed as

2 $$\begin{aligned} I_\text {PL}(\vec {k}) \propto \sum _{\vec {R}, z} \left| {\mathscr {F}} \left\{ {\hat{P}}_{t-z} V_{\text {TP}}(\vec {r}-\vec {R}) {\hat{P}}_z \psi _0(\vec {r}) \right\} \right| ^2, \end{aligned}$$

where $\psi _0(\vec {r})$ is the incident wave function and ${\hat{P}}_{z}$ is an operator representing the frozen-phonon propagation through the specimen. The sum goes over all positions in the specimen and *t* is the specimen thickness. If ${\hat{P}}_{z}$ and the multiplication with $V_{\text {TP}}(\vec {r}-\vec {R})$ commuted, Eq. (2) would take the form

3 $$\begin{aligned} I_\text {PL}(\vec {k}) = \left( 1-\exp \left[ - t/\lambda \right] \right) \left| V_{\text {TP}}(\vec {k}) \right| ^2 \otimes \left| {\hat{P}}_{t} \psi _0(\vec {k}) \right| ^2, \end{aligned}$$

with $\otimes$ denoting convolution and $\lambda$ the MFP of plasmon excitation. This means that the plasmon-loss diffraction pattern would indeed be nothing but the zero-loss pattern convolved with the square of the transition potential. However, the two terms do not commute strictly, because the Fresnel propagation, which is part of ${\hat{P}}_{z}$, is represented by a convolution in real space, which does not commute with a real space multiplication.

**Figure 5:**
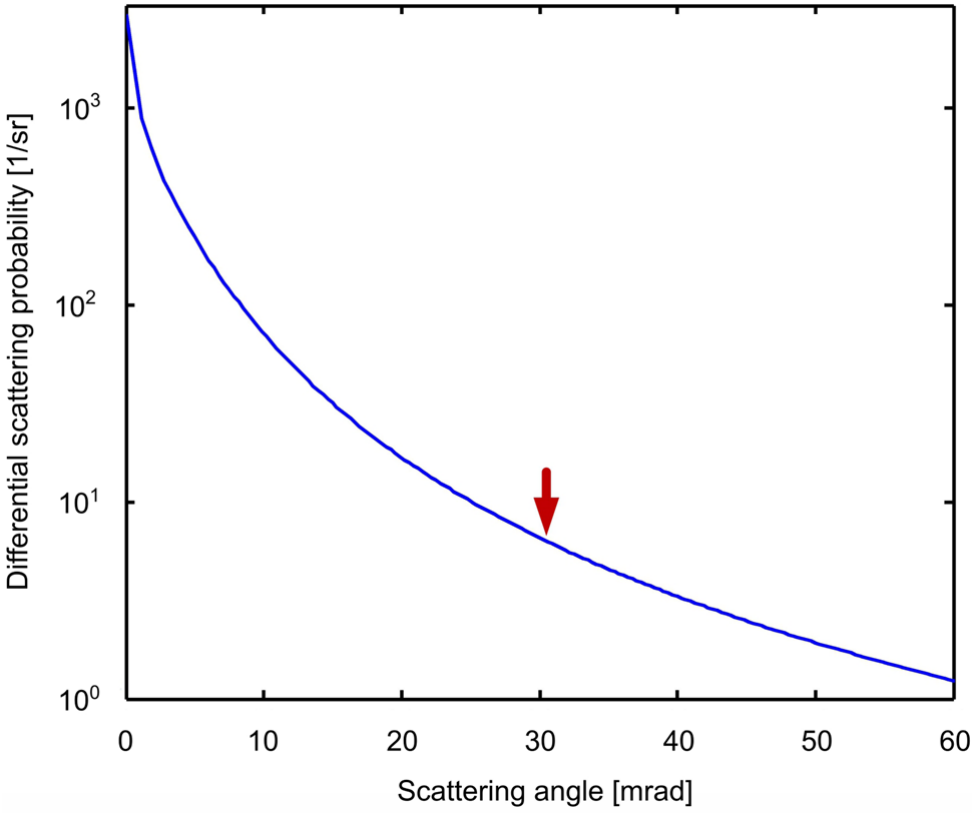
The differential plasmon excitation probability $\left| V_{\text {TP}}(\vec {k})\right| ^2$ according to Eq. (1). Up to a scattering angle of $~30~\mathrm {mrad}$ it drops by five orders of magnitude, as marked by the red arrow.

**Figure 6:**
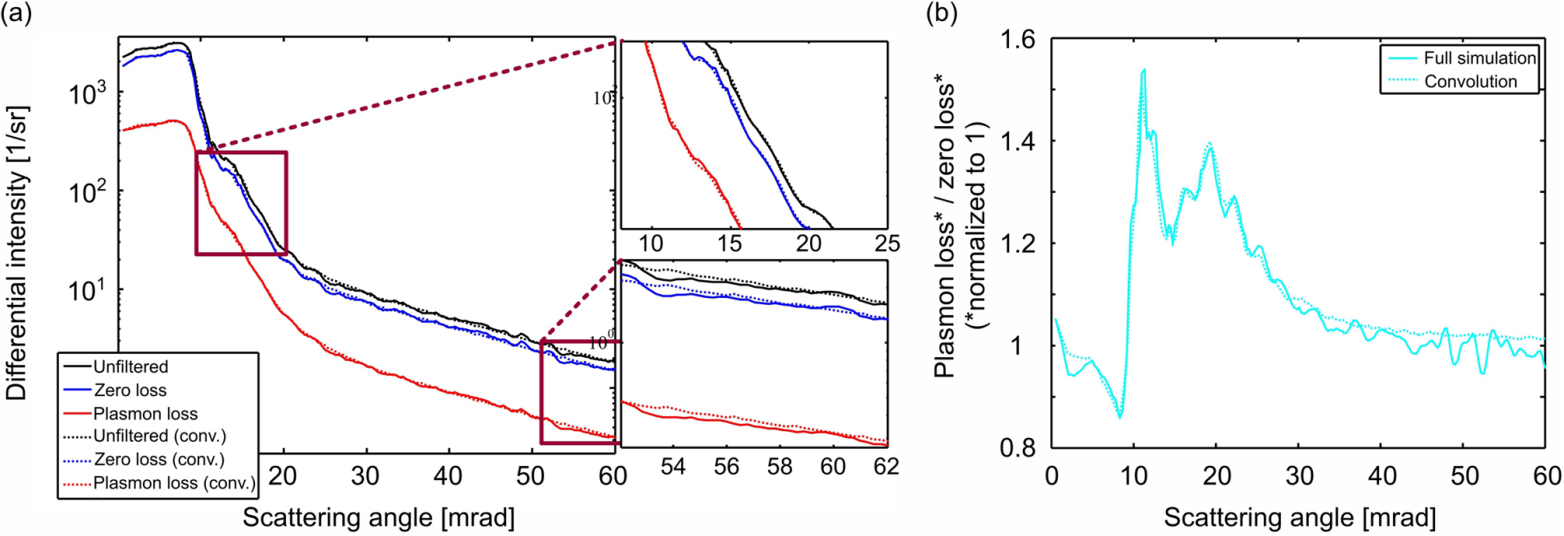
Approximation of the plasmon-excitation simulation by diffraction pattern convolution: (**a**) displays the result of the convolution calculation in Eq. (3) in comparison with the results of the full simulation as represented by Eq. (2). In (**b**) the normalised ratio between zero-loss and plasmon-loss signal is shown for both methods. Corresponding to the smallest thickness in Fig. 3, the calculations are done for [100] Si of $30\,\mathrm {nm}$ thickness. The convolution is able to approximate the full simulation with high accuracy for the angle range of 0–$35\,\mathrm {mrad}$ as can be seen in (**b**). This is remarkable, because the scattering probability has dropped up to five magnitudes for this scattering angle as marked in Fig. 5.

Nevertheless, $V_{\text {TP}}(\vec {k})$ is very sharply peaked in reciprocal space, as can be seen in Fig. 5, and hence wide in real space. This does allow the use of Eq. (3) at least as an approximation. The applicability is demonstrated in Fig. 6, where the results of the convolution (dashed lines) are displayed alongside the results of the full simulation (solid lines). The differential intensities (a) as well as the normalized plasmon-loss/zero-loss ratio (b), show an exceptional agreement with the full simulation in the investigated angular range. This confirms the approximative applicability of Eq. (3) for the inclusion of plasmon excitations into simulations. This can be very advantageous, since the simulation according to Eq. (2) is much more demanding, because each summand requires one frozen-phonon multislice simulation. For Eq. (3) on the other hand, a single traditional frozen-phonon calculation suffices and only one convolution is added, resulting in negligible change of computational effort.

Furthermore, this gives a certain insight into the origin of the redistribution: The rather steep maximum of $\left| V_{\text {TP}}(\vec {k})\right| ^2$ for very small scattering angles suggests that the convolution in Eq. (3) will primarily only cause a smearing of intensity by a few multiples of the characteristic angle, i.e less than 1 mrad. For a scattering angle of $~20\,\mathrm {mrad}$ for example, the scattering probability is five orders of magnitude smaller than for scattering without direction change. Although this smearing is happening and is causing the plasmon-loss diffraction patterns in Fig. 1 to be blurred as discussed, it is not the reason for the intensity redistribution into the low-angle range: If the transition potential was limited to few $\mathrm {mrad}$, neither the simulations nor the convolution could reproduce that. The elevation between 10 and $~30\,\mathrm {mrad}$ seen in Fig. 6 is caused by the very small tail of the transition potential, as similarly suggested in Ref.^12^. Its small magnitude for the corresponding scattering is counteracted by the fact that the diffracted intensity itself in this angular range is in part more than three orders of magnitude smaller than that in the center, meaning that even a redistribution of only $~10^{-5}$ from the $~10^3$ times brighter central beam can still account for several percent increase as observed. This does in turn mean that the scattering processes causing the observed plasmon influence are only a very small fraction of the overall plasmon excitation events.

Finally, while a convolution does redistribute the intensity, the symmetry of the diffraction pattern is not affected by the convolution as long as the center of mass of $\left| V_{\text {TP}}(\vec {k})\right| ^2$ itself is at $\vec {k} = (0, 0)$, which is the case here. Therefore, the results of first moment measurements in momentum resolved STEM, can be expected not to be influenced by plasmon excitations, although the low-angle range is evaluated. This is true, as long as Eq. (3) holds, which should be the case especially for the thin specimens used to measure atomic scale electric fields. The actual impact on the experimental data acquired will be investigated in a subsequent section.

### Spatially resolved evaluation of the scattering

4D STEM allows for the investigation of the momentum space, while keeping the atomic spatial resolution of the aberration-corrected probe, which will be exploited in the three examples presented in the following. In the first example, we will show how potentially present sample drift can be corrected for. Secondly, we will use the data sets to generate synthetic images to investigate the influence of inelastic scattering on the most common STEM technique, i.e. (HA)ADF imaging, and how the ratio of inelastic/elastic scattering changes with respect to the positions of the atomic columns. Finally, we will elucidate the impact of inelastic scattering on first moment STEM imaging^47^.

#### Consideration of sample drift

**Figure 7:**
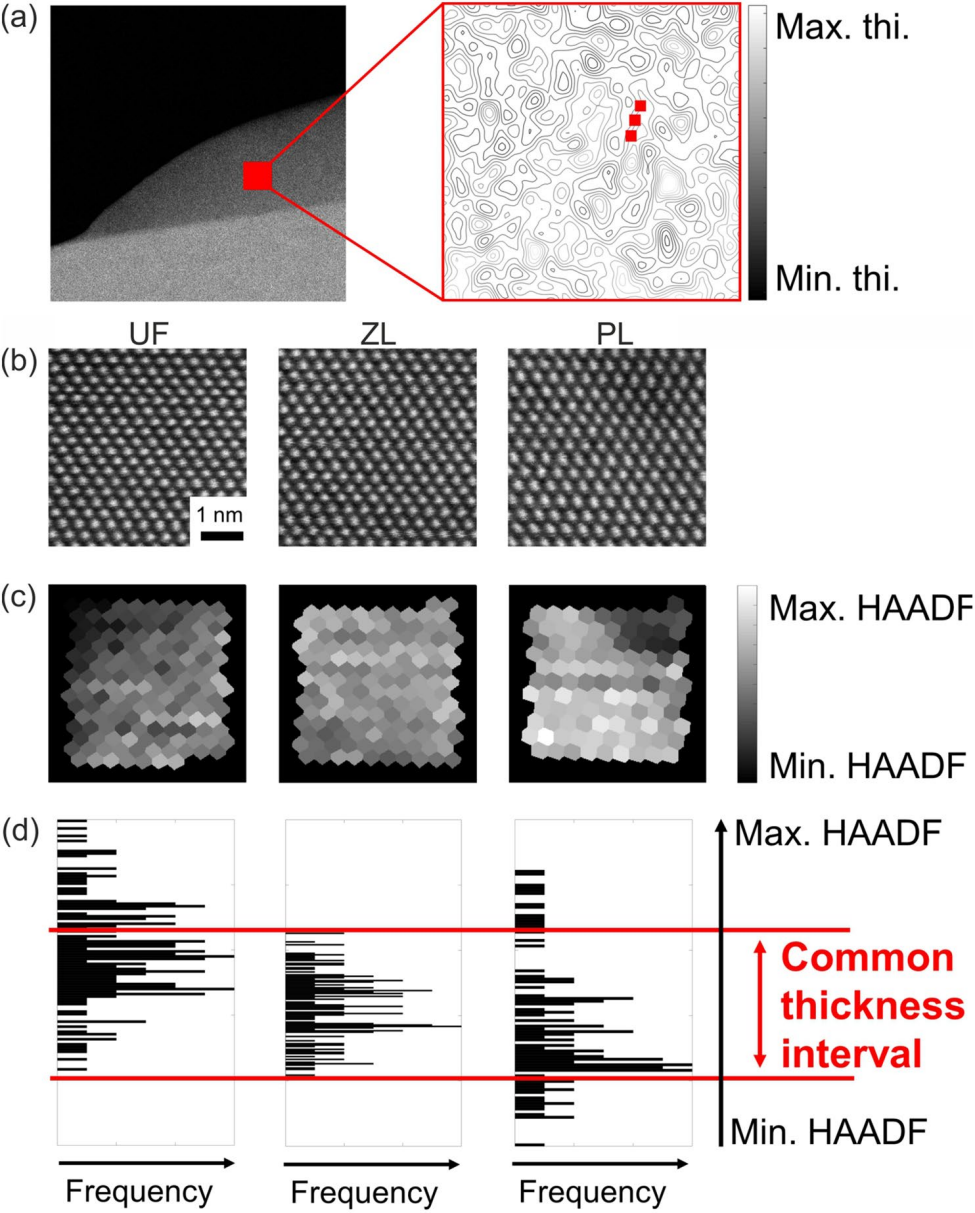
The sample drift is accounted for by retrieving a common thickness interval between the recordings. (**a**) HAADF image of the Pt [110] specimen with intensity contours illustrating the local variations in thickness. Three possible points of acquisition are highlighted and all contain atomic columns from a same thickness isoline. (**b**) Unfiltered HAADF images for each recording. The variation in thickness from one recording to another is measured using (**c**) a Voronoi analysis which allows to find the (**d**) thickness interval common to the three recordings and finally make sure that further analysis are done using Voronoi-integrated diffraction patterns only from this common thickness interval.

We use the Pt case to exemplify the impact of specimen drift. Although the measurement did not suffer from severe sample drift, changing the settings of the energy filter and, more importantly, the acquisition itself inevitably cause delays of several minutes in between the acquisitions employing different energy windows, during which the specimen might have drifted to a position with a slightly different thickness. In addition to that, regions present in the recordings might suffer from thinning due to knock-on damage or deposition of contaminations. This means even without considering the drift, the thickness might not be conserved from one energy window to another, such that the differences between different energy settings might in fact be caused by thickness gradients. Finally, it is worth noting that the Pt specimen displayed thickness gradients significantly more pronounced than the Si ones, due to the different preparation schemes used. We nevertheless demonstrate that the differences in the observed characteristic angular dependencies of elastic and inelastic scattering are not affected by a varying specimen thickness among the three data sets, as summarized schematically in Fig. 7a.

To this end, we use the signal of an ADF detector mounted in front of the energy filter which is thus invariant against its different settings, and whose signal is recorded simultaneously with all data sets. In each of the atomically-resolved STEM images for the three energy windows depicted in Fig. 7b, we performed a Voronoi segmentation, as described in Ref.^33^, to obtain the integral Voronoi intensities in Fig. 7c which clearly exhibit thickness variations in all three scans, potentially coming both from drift and thickness change. The histograms in Fig. 7d assess the existence of a common thickness interval. By exclusively using results included in that interval, we can thus perform a comparison of energy-dependent scattering characteristics using exactly the same specimen thicknesses.

#### Influence of inelastic scattering on ADF imaging

**Figure 8:**
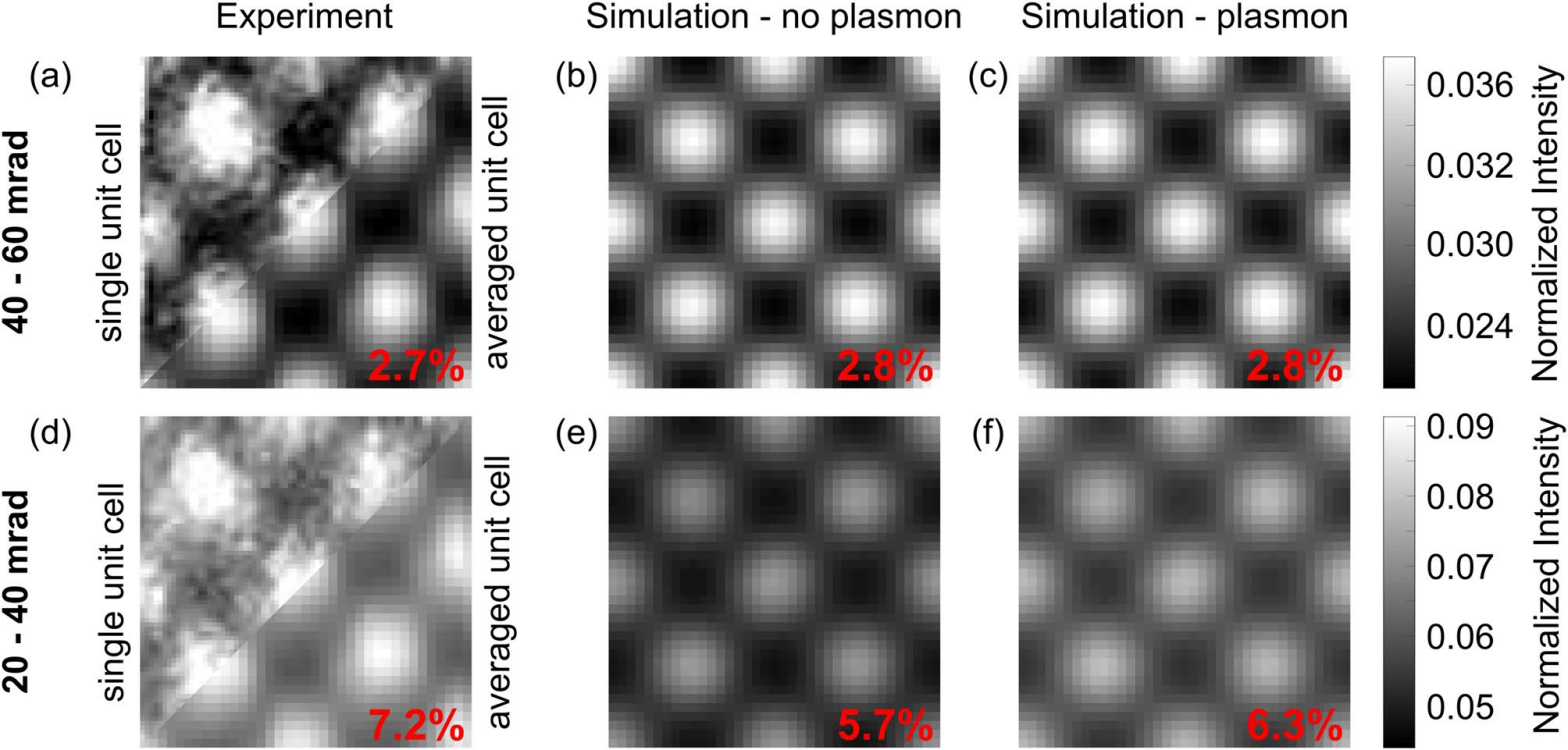
Comparison of experimental and simulated ADF intensities of Si neglecting and including single plasmon excitation, respectively. The ADF images generated from the three data sets are shown in (**a**–**c**) for an angular range of 40–60 mrad and in (**d**–**f**) for 20–40 mrad, respectively. While both simulations describe the higher-angle regime accurately, the low-angle regime is better reproduced by the simulation including plasmon excitation. The average intensities of the unit cells given in fractions of the impinging beam are indicated in the individual images.

The comparison of Figs. 3 and 4 shows that the adopted simulations reproduce the angular dependencies of zero-loss and plasmon-loss signal qualitatively correctly. If the description of the experiment indeed is improved by including plasmon excitation, this has to be reflected in the ADF images generated from both experiment and simulation for the Si material depicted in Fig. 8. The images are generated by summing the intensity in a distinct annular range of the unfiltered data set and plotting this value with respect to the scan position. For reasons of clarity, only an area of one unit cell of the bigger field-of-view image is shown in Fig. 8a–c utilizing an angular range from 40–60 mrad, where inelastic scattering can be expected to have only little effect. The top left part of Fig. 8a is the central unit cell of the image, whereas for the lower right part the $\sim \,50$ individual unit cells present in the whole image are averaged to achieve a higher signal-to-noise ratio according to Ref.^30^. Figure 8b,c depict the images derived from the simulations neglecting and taking into account plasmon excitation, respectively. To accurately resemble the experiment, the experimental MTF and partial spatial coherence are accounted for in the simulations. Again experiment and simulation share a common intensity scale for direct comparison. The average intensities of the unit cells given in fractions of the impinging beam are indicated in the individual images. For this higher angle range all three images are in very good agreement, as expected from the angular distributions shown in Figs. 3 and 4. The average values of the simulated unit cells, which both are 2.8% of the impinging beams intensity, agree very well with the one of the experimental image, which is 2.7%.

The analogue figures for the comparison at the lower angle regime, i.e. 20–40 mrad, are arranged in Fig. 8d–f. The non-plasmon simulation in Fig. 8e significantly underestimates the experimental intensity shown in Fig. 8d, i.e. 5.7% versus 7.2%. Including single plasmon excitation as depicted in Fig. 8f, the simulated intensity is increased to 6.3%, which leads to a closer resemblance of the experimental intensity. Comparing Fig. 8d,f, it becomes apparent that the background intensity, i.e. the intensity between the atomic columns, is higher in the simulations which include plasmon excitation. This elevated background intensity becomes obvious in the experimental images as well, e.g. when the two angular ranges depicted in Fig. 8a,d are compared. The position dependence of the inelastic scattering will be discussed in more detail in the subsequent section. Anyways, the improved fit of the simulation including plasmon scattering highlights the necessity to consider plasmon excitations in all STEM techniques to which this angular range is contributing, e.g. low-angle ADF (LAADF), angle-resolved STEM (ARSTEM)^12^ or momentum resolved STEM, which will be discussed in a subsequent section of this manuscript.

However, the simulated intensity is still too low. Possible explanations for this discrepancy could be experimental uncertainties, e.g. the sample thickness, which is determined by PACBED, whose accuracy is in the range of a few nm^32^. Another factor could be the small mistilt of $\sim$ 2 mrad observed which could influence the diffraction conditions, e.g. the positions of interference fringes, and therefore affect the ratio of elastic to inelastic scattering. Furthermore, the excitation of a second plasmon neglected in the simulation could influence the experimental intensity. This is rather unlikely, since the second plasmon peak is not very pronounced as can bee seen in the corresponding EEL spectrum depicted in Fig. 2a. More interestingly, there are other factors aside from plasmons which contribute to the low angle scattering as well, which are still neglected in the simulation. These could e.g. be that uncorrelated phonons are used for the simulation^18,20,25^, the presence of amorphous layers from sample preparation^29–31^ or the usage of isolated atom scattering potentials which e.g. neglect bonding effects^13,26–28^. All of these effects have been shown to influence the intensity at low scattering angles as well. In total, however, we have shown that plasmon excitations play a major role.

**Figure 9:**
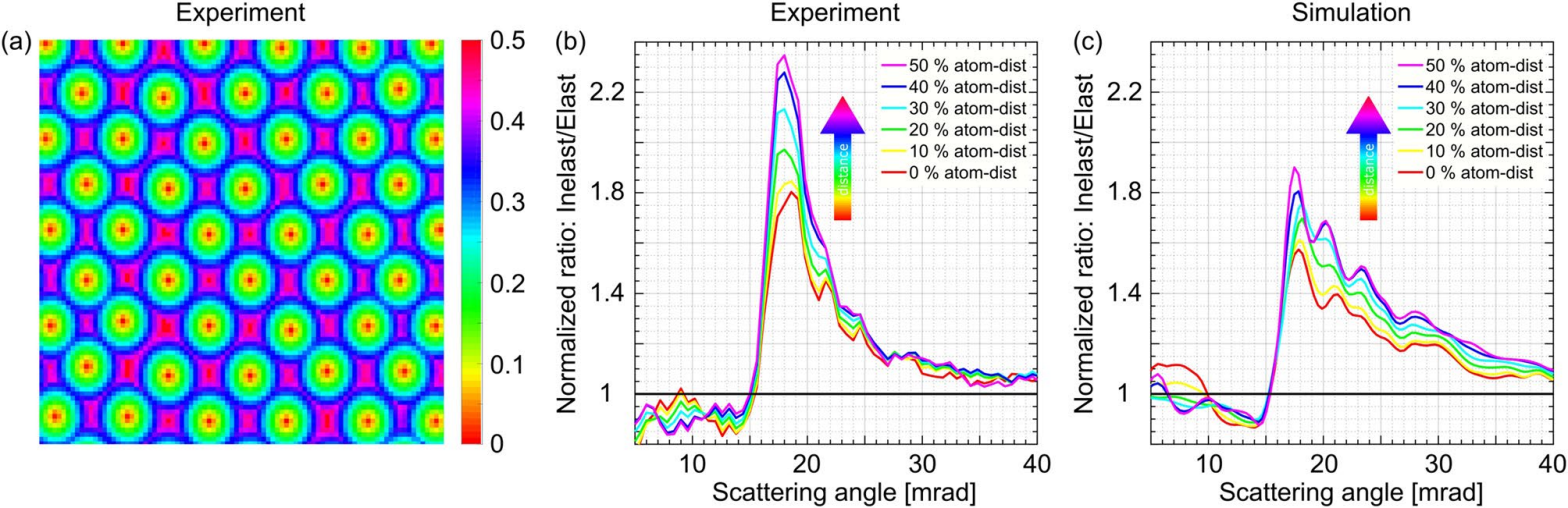
(**a**) Distances to atomic column centers in units of the average atomic distance. (**b**) Experimental ratios after a normalisation of both plasmon- and zero-loss signals for different distances to the atom column center. (**c**) Same ratio as shown in (**b**) for the simulated signals.

The recorded data allows to investigate the effect of inelastic scattering on sub-unit-cell length-scale. To this end, the atomic column positions are found in the HAADF images and for each pixel in the image, the distance to the nearest atomic column is calculated in units of the average atomic distance. Fig. 9a shows a magnified example of a 2D map with color-coded distances for the Si [010] sample. Fig. 9b shows the ratio of the normalized signals for plasmon-loss and zero-loss intensities averaged over all pixels with the same distance to the atom column center. With increasing distance from the center of atom columns, the ratio increases outside the semi-convergence angle. It can be explained by the fact that elastic or quasi-elastic scattering, such as thermal diffuse scattering, is enhanced on column positions, whereas the probability of plasmon excitation is uniformly distributed over the unit cell to a good approximation. Consequently, the ratios in Fig. 9b are increased with increasing distance to the columns. While the absolute plasmon related intensity does not change significantly, its relative contribution rises. Figure 9c shows the same ratio as in Fig. 9b but for the simulated signals. The observable tendencies are well reproduced by the simulations, i.e. the ratio above the semi-convergence angle is decreased relative to the unit-cell average directly on an atomic column, and it is increased relative to the unit-cell average between the columns. This explains the apparently elevated background intensity visible in Fig. 8d,f.

#### Influence of inelastic scattering on first moment imaging

**Figure 10:**
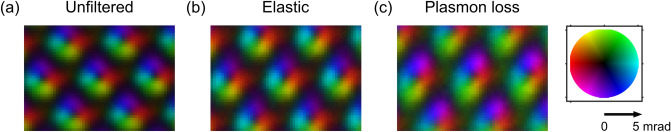
Results of first-moment imaging from the thicker region of the Pt sample. The signals generated from the unfiltered, zero-loss and plasmon-loss data are shown in (**a**–**c**), respectively. In analogy to Fig. 8, averaged unit cells are generated from a bigger field of view image to improve the signal-to-noise ratio.

Figure 10 displays the first-moment images generated from the 4D data sets of the thicker region of the Pt sample. The unfiltered image is shown in Fig. 10a, while (b) and (c) depict the zero-loss and plasmon-loss data, respectively. Like in Fig. 8, averaged unit cells generated from a bigger field of view image are shown. The color code is kept the same for all three images. Qualitatively, the images show the same features, i.e. rings of continuously changing color around the atomic columns. This reflects the attraction of the impinging probe towards the atomic columns due to Coulomb interaction. The elongation observable along the diagonal of the plasmon-signal image (Fig. 10c) is most likely caused by drift or residual astigmatism, which is visible as well in the corresponding ADF image depicted in Fig. 7b. Quantitatively, there are slight differences between the individual data sets, e.g. the values of maximum are 3.75 mrad for the unfiltered case, 4.3 mrad for the zero-loss case and 5.08 mrad for the plasmon-loss case. However, these discrepancies can be regarded as negligible considering the noise present in the data. This means that plasmon excitations do not significantly influence the first-moment signal, as expected from the theoretical considerations presented in the simulation section.

## Conclusions

We measured the angular distribution of the electron scattering at different energy losses for two different materials. In total, we have shown that plasmon excitations significantly influence low angle STEM intensities ($\sim$ 0–40 mrad). An angular range in which other impacts have been discussed before, i.e. correlated phonons, atomic scattering potentials or the presence of amorphous layers. In the case of inelastic scattering, intensity is redistributed from the central beam towards the outer region. The influence of the inelastic scattering in the low-angle regime increases with rising thickness and the relative impact is weaker on an atomic column compared to the positions between the columns. These are very general features, since we found the analogue behaviour for two complementary materials (Si and Pt) using two different microscopes operating at very different experimental conditions: incident beam energies (200 kV vs. 300 kV) and semi-convergence angles (9 vs. 15.1 mrad). While the high-angle regime is hardly affected, since the angular distributions of elastic and inelastic scattering are identical there, plasmon excitations affect all STEM methods involving low scattering angles, e.g. bright field (BF), annular BF (ABF), ARSTEM or LAADF imaging. Due to symmetry, first-moment STEM measurements are not significantly affected, although they involve low scattering angles. Therefore, plasmon excitations have to be considered in simulations to allow for a quantitative evaluation of experimental data. A potential route for the implementation into the multislice algorithm is outlined in this study. The simulations carried out using the adopted STEMsim code qualitatively resemble the experimentally observed intensity redistribution very well. The experimental angular, thickness and spatial dependencies are well reproduced by the simulations. Moreover, the good description of the experimental data using a convolution approximation is very promising, since it could allow for the implementation of plasmon excitations into multislice simulations without significant increase of computational time.

## Methods

For the Si, two cross-sectional TEM lamella-type specimen were prepared in [010]-direction using a dual beam focused ion-beam scanning electron microscope (FIB-SEM) machine (JEOL JIB 4601F). To achieve reproducible measurements with the same sample thicknesses, the first sample (Si sample A) was deliberately prepared with thickness gradient increasing from the tip of lamella to its bottom and the second sample (Si sample B) was prepared with steps of defined thicknesses. The Ga ion beam energy was gradually decreased from 30 to 5 keV to limit the amorphous layers introduced at higher energies^48^. Using a NanoMill (model 1040, E. A. Fischione Instruments, Inc., Export, PA, United States), final Ar ion polishing was performed with milling energies of 900 eV and subsequently 500 eV with an inclination angle of 10 degrees with respect to the sample surface^49^. Finally, sample A exhibits a thickness gradient with a lowest thickness of $\sim$ 20 nm and the second one includes steps of 30, 55, 85, 110 and 135 nm thickness. Thicknesses were determined by comparing the PACBED measurements carried out separately under optimized conditions using a FEI Titan 80/300 and complementary image simulations detailed in the next paragraph. In addition, a cross-sectional TEM lamella of Pt was prepared in [110]-direction with a FIB-SEM instrument (FEI Helios NanoLab 400S), using the conventional lift-out method. A continuous thickness gradient was introduced at the tip of the lamella by using irradiation of Ga ions with 5 keV energy. Polishing was done using Ar ions in a NanoMill machine with milling energies of 900 and 500 keV, using an inclination angle of 10 degrees. To remove contaminations, plasma cleaning of the samples was performed prior to inserting them into the microscope (model 1020 E. A. Fischione Instruments, Inc., Export, PA, United States).

Energy filtered diffraction patterns from Si sample A as well as quantitative HAADF STEM from Si sample A and B were performed with a FEI Titan 80/300 TEM working at 300 kV with 9 mrad semi-convergence angle and a spherical aberration of 1.2 mm. Diffraction patterns were recorded while scanning the specimen. Energy filtering was performed using a Gatan GIF with a 10 eV slit around 0 eV (zero loss) and 16 eV (plasmon loss). Quantitative STEM was performed with a Fischione 3000 ADF detector under conditions described previously^33^. Frozen-phonon multislice simulations employing the Einstein model with uncorrelated phonons were calculated for quantitative comparison to the experimental image intensities using the STEMsim code^19^. A fully detailed description of the thickness determination by quantitative HAADF STEM used here can be found in Ref.^33^. For thickness determination from PACBED, the inner part of the experimental PACBEDs up to approximately 20 mrad were compared to thickness-dependent simulations^31^ performed in STEMsim^19^.

The Si sample A and Pt were also investigated by a double $C_S$-corrected JEOL JEM2200FS (Jeol Ltd., Tokyo, Japan) operating at 200 kV. Here, a sub Angstrom probe size is achieved allowing for atomic spatial resolution. During the measurements, the hexapoles of the image corrector were switched off in order to avoid the cut-off and distortion at the wider angular ranges of diffraction patterns^50^. The condenser aperture used leads to an probe semi-convergence angle of 15.1 mrad. The combination of this aperture and the spot size setting of 10C results in a comparably low beam current of 2.37 pA.

An in-column omega energy filter allows to obtain energy filtered diffraction patterns after the interaction of the electrons and the specimen. Using an adjustable slit at the exit plane of the energy filter, different energy windows were chosen, i.e. no slit, − 5 to 5 eV and 11–21 eV for unfiltered, zero-loss filtered and plasmon-loss measurements, respectively.

A pnCCD-based, fast direct single electron imaging detector was used to acquire the convergent beam electron diffraction (CBED) patterns at every scanning position for scattering angels up to 60 mrad. Calibration of sampling of the pnCCD detector was done by measuring the known radius of the direct beam and was further confirmed by measuring the radius of the first order Laue zone of Si. A 4 $\times$ 4 nm field of view of the specimen was scanned, resulting in a diffraction pattern at each of the 256 $\times$ 256 probe positions. The standard full frame readout of the camera with 1000 frames per second is used, i.e. every CBED image is recorded with an image area of 264 $\times$ 264 pixels^7,11^. For quantitative analysis the normalized CBED patterns are achieved by dividing every CBED by the beam intensity, i.e. recorded image of the probe at a position with no specimen on the pnCCD at the same conditions as the data sets. To be able to compare the angular dependency of scattering at every energy range, the PACBED images of different energy windows are aligned by calculating the center of mass of the images. The acquisition of each of the measurements at different energy windows takes around 65 seconds. During this period, sample drift could be present which could result in different sample thicknesses for unfiltered, zero-loss and plasmon-loss measurements. To prove that there is no significant drift during the measurements, a second unfiltered data set was measured after the acquisition of zero-loss and plasmon-loss measurements and compared to the one before.

The simulations including plasmon excitations according to the scheme described in the article were conducted within the adapted STEMsim software package^19^. The numerical grid of the frozen-phonon simulations was $1408\times 1408$ pixels and $11\times 11$ unitcells were used laterally to form the super cell with the respective thickness. Atomic scattering amplitudes were taken from Ref.^51^ and the Si lattice constant of $5.4\,\AA$ was chosen as the slice thickness. Mean free paths for the plasmon interaction were calculated according to Ref.^52^. Per slice 90 different positions for the plasmon transition potential were used.
